# Supplementary figures and images for: Exploratory trajectory inference reveals convergent lineages for CD8 T cells in chronic LCMV infection
Source: PLoS One. 2025 Sep 19;20(9):e0332406. doi: 10.1371/journal.pone.0332406 (PMC12448961; doi:10.1371/journal.pone.0332406)

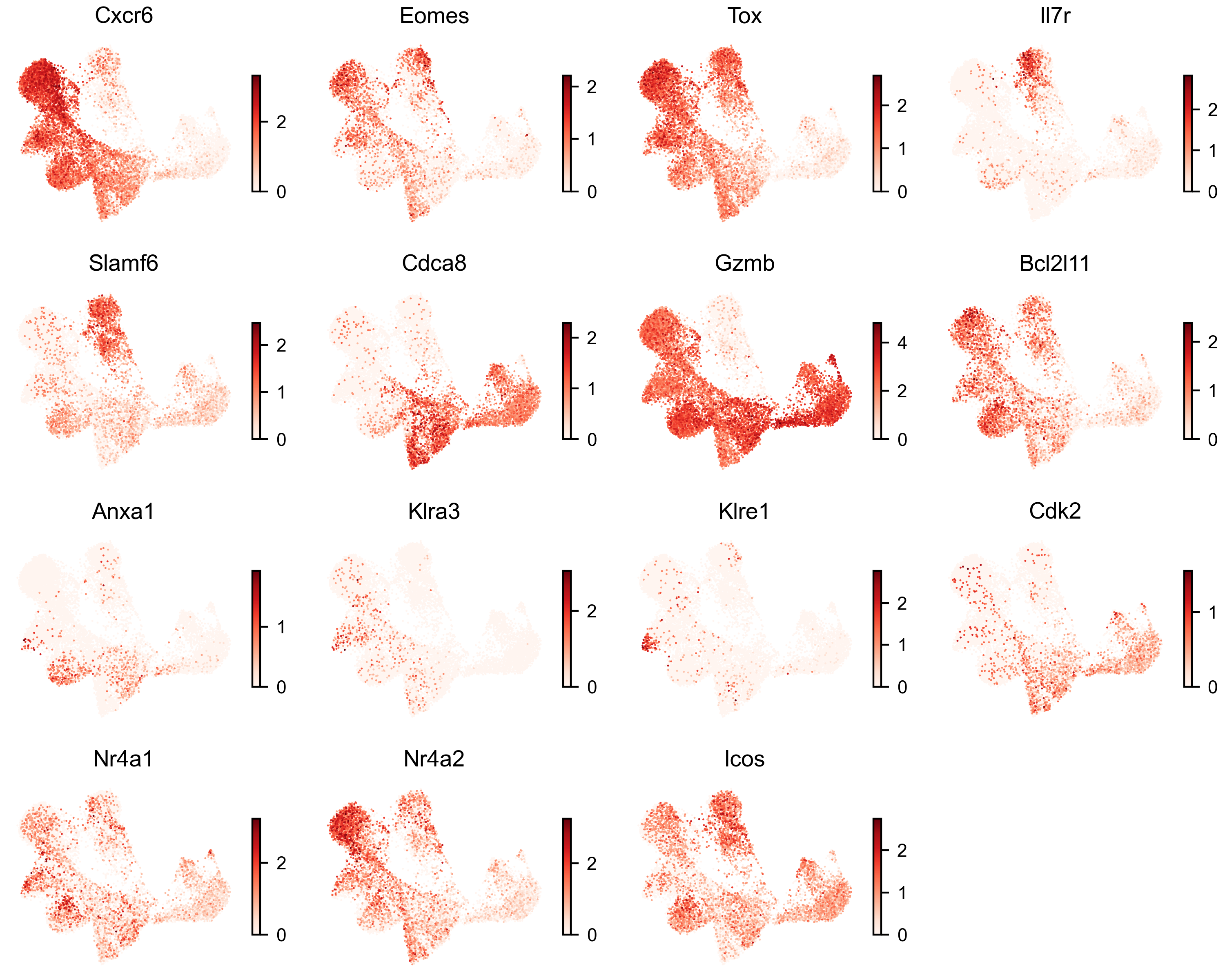

Supplement: S1 Fig — (TIF) [file pone.0332406.s001.tif]

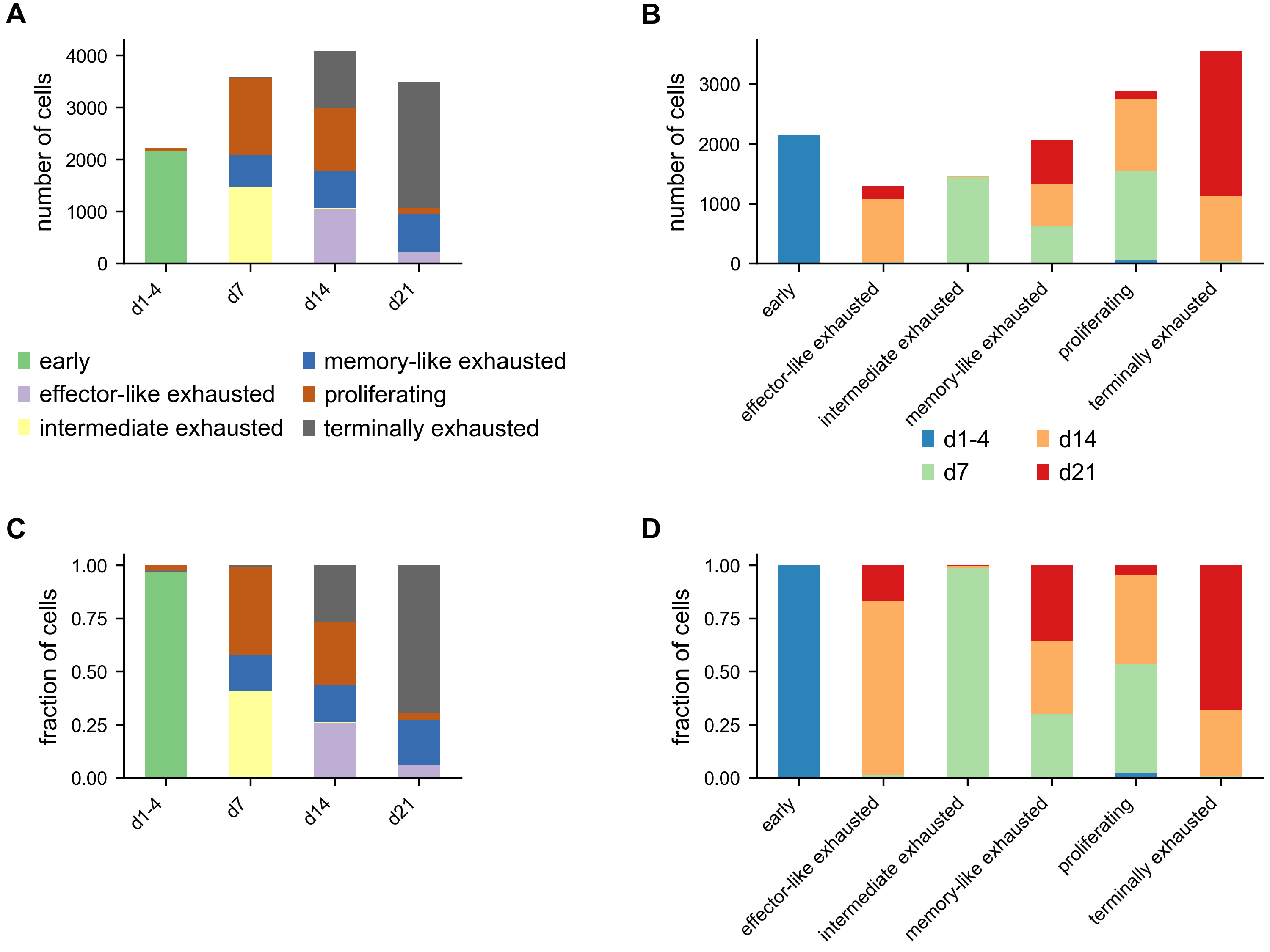

Supplement: S2 Fig — (A) Cell numbers per group for each sample time point. (B) Cell numbers per sample time point for each phenotypic group. (C) Fractional composition per sample time-point. (D) Fractional composition per phenotypic group. (TIF) [file pone.0332406.s002.tif]

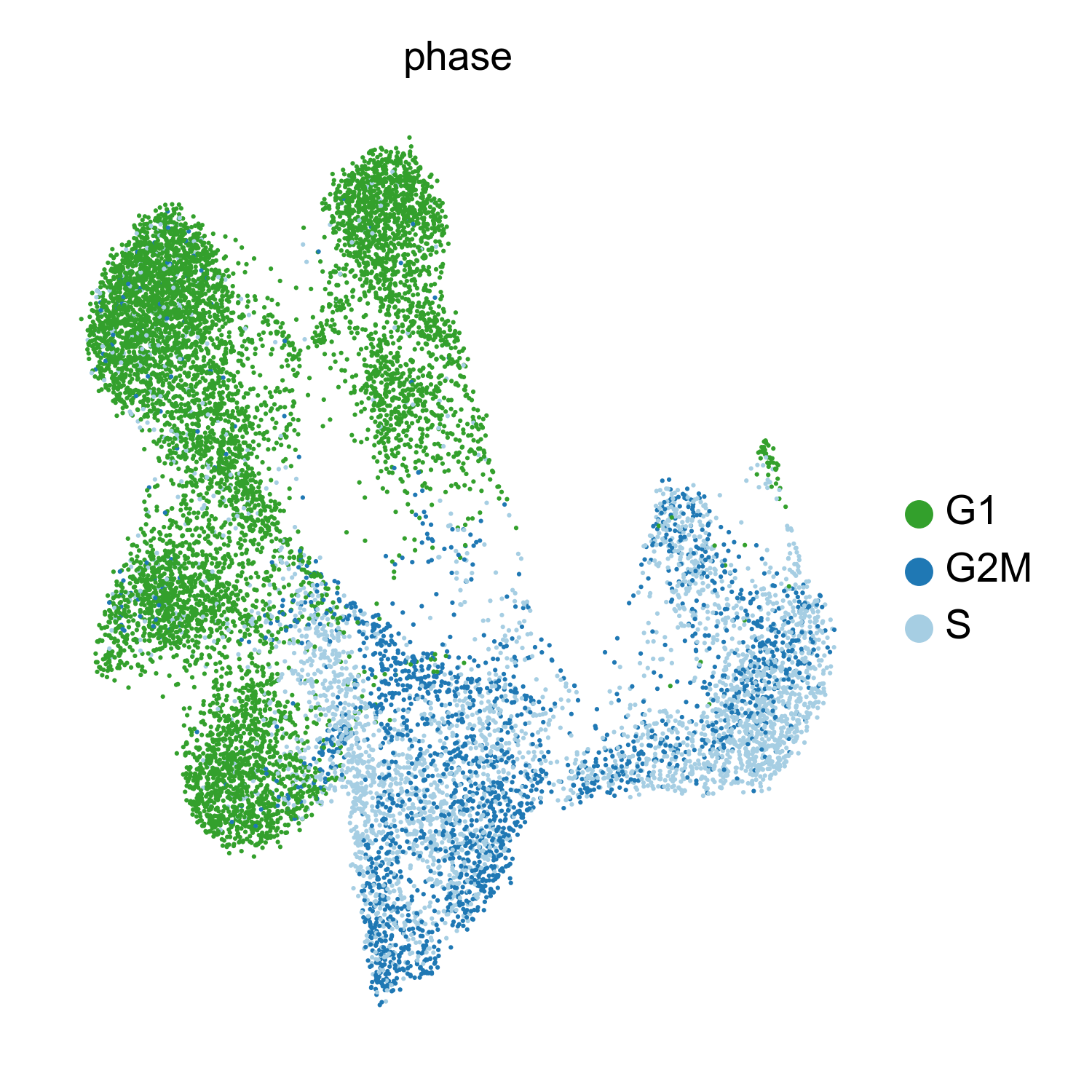

Supplement: S3 Fig — The early and proliferating subpopulations were classified as a mix of G2/M- and S-phase cells. (TIF) [file pone.0332406.s003.tif]

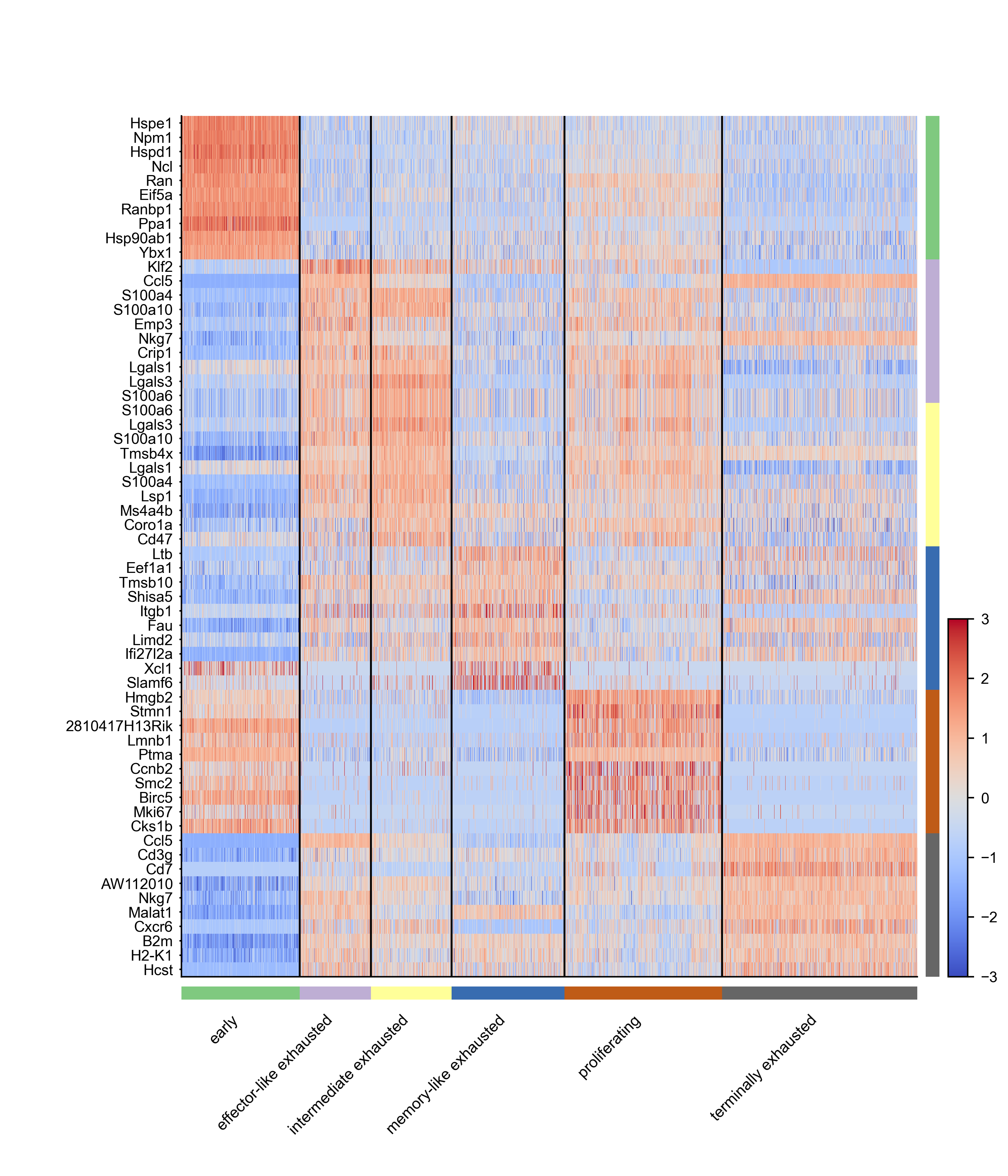

Supplement: S4 Fig — (TIF) [file pone.0332406.s004.tif]

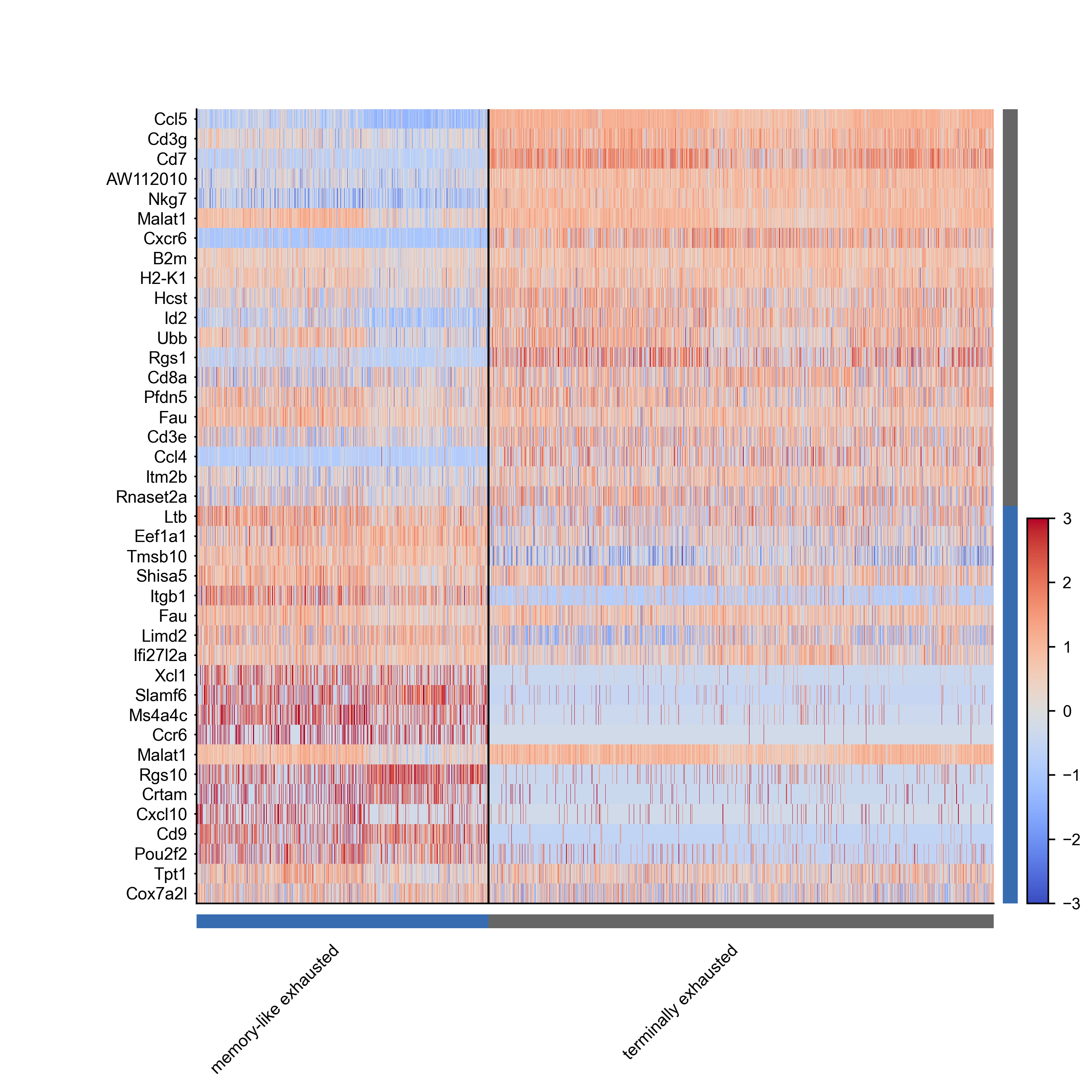

Supplement: S5 Fig — (TIF) [file pone.0332406.s005.tif]

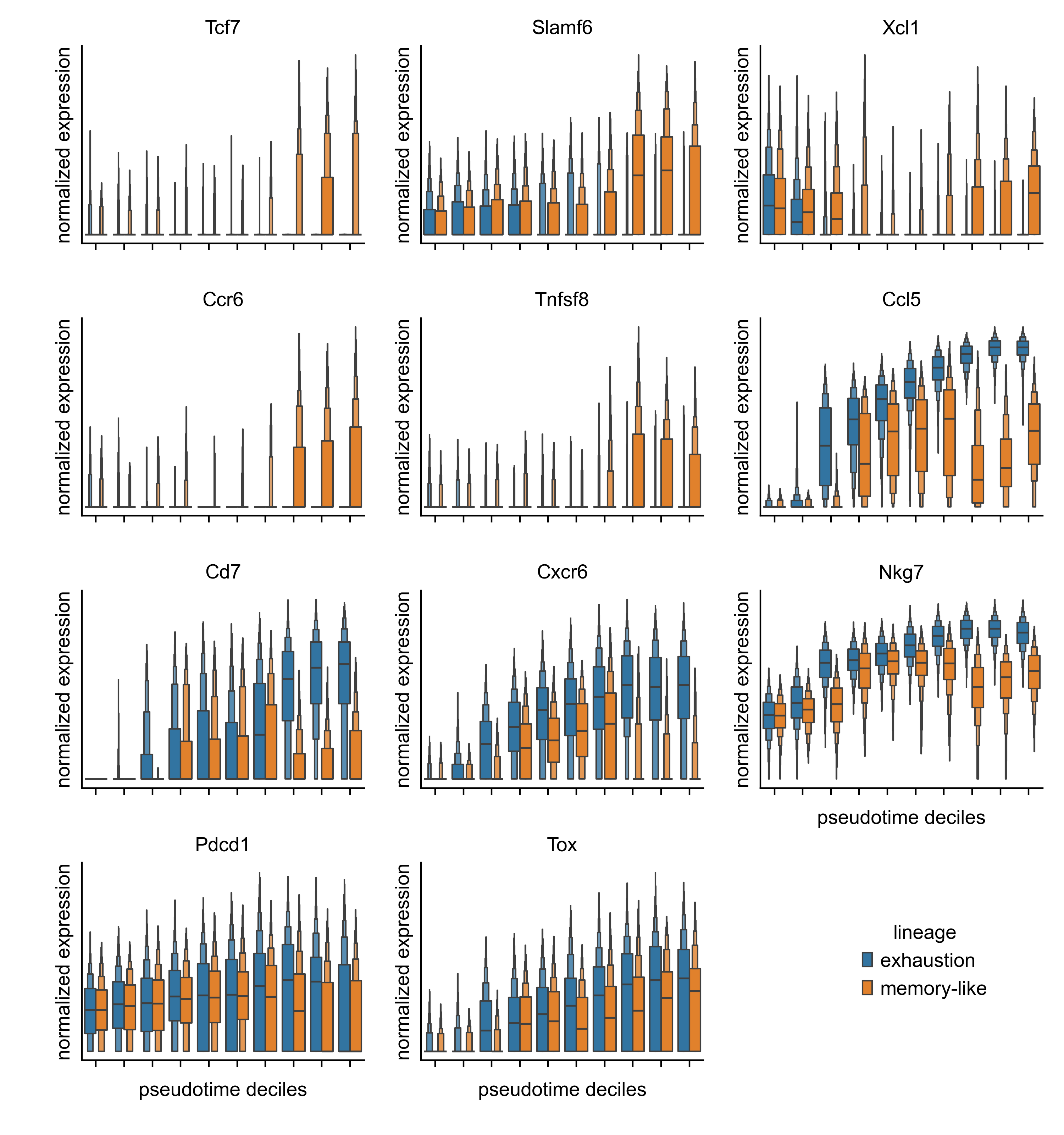

Supplement: S6 Fig — Genes with differential expression patterns between exhausted and memory-like trajectories are shown. (TIF) [file pone.0332406.s006.tif]

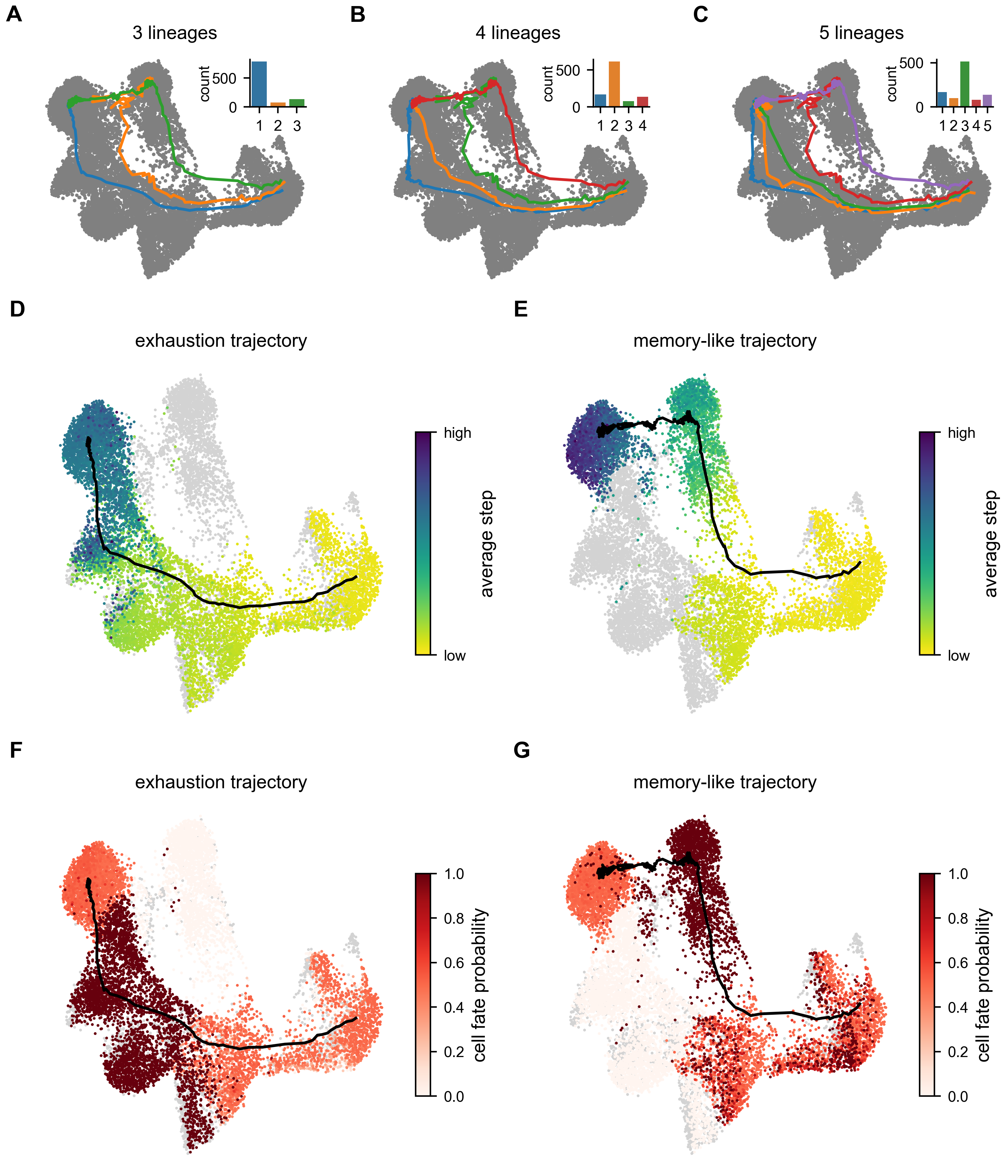

Supplement: S7 Fig — (A–C) Three, four, and five lineages are obtained by cutting the hierarchical tree at the corresponding height. Insets show the number of simulations per lineage. Each lineage is shown in a distinct color. (D-E) Average step for cells in the exhausted (D) and memory-like trajectory (E). (F–G) Cell fate probabilities along the exhausted (F) and memory-like trajectory (G). (TIF) [file pone.0332406.s007.tif]

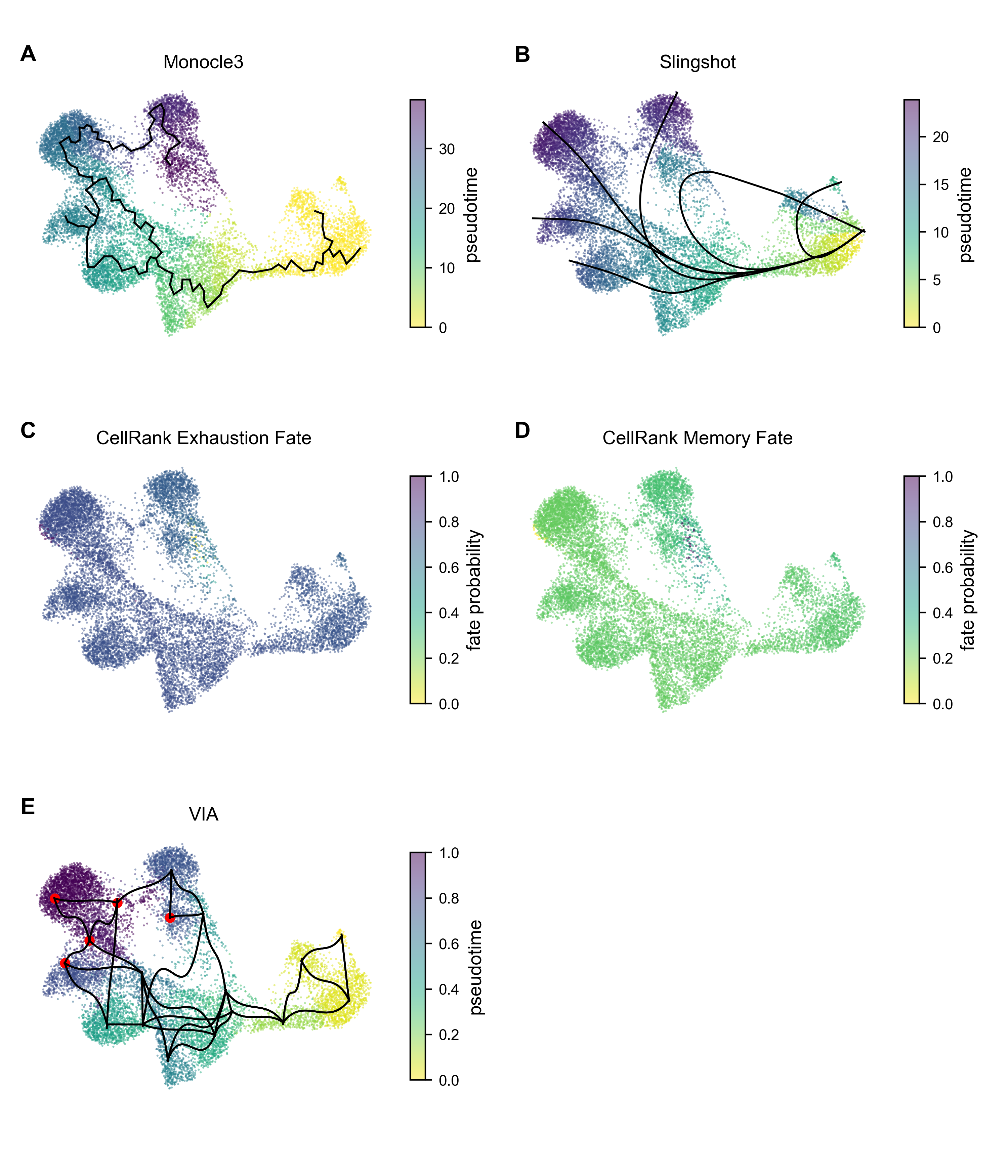

Supplement: S8 Fig — (A) Trajectories and pseudotime inferred by Monocle3 [9,23]. (B) Trajectories and pseudotime inferred by Slingshot [8]. (C–D) CellRank [6] fate probabilities for the terminally exhausted (C) and memory-like endpoint (D). (E) Trajectories and pseudotime inferred by VIA [24]. (TIF) [file pone.0332406.s008.tif]

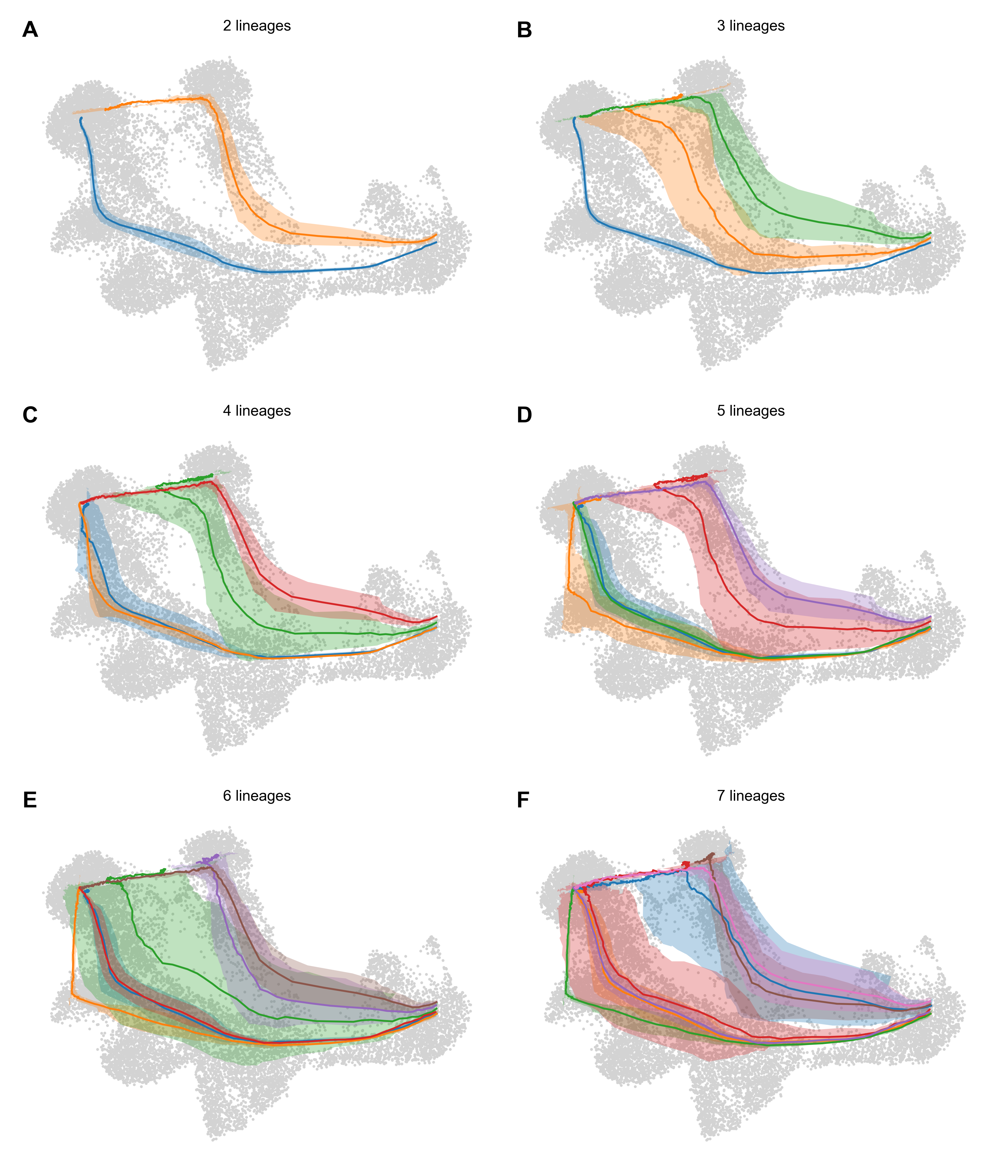

Supplement: S9 Fig — (A–F) Mean and standard deviation of inferred trajectory coordinates across 10 runs for different numbers of inferred lineages. Each lineage is represented by a distinct color. (TIF) [file pone.0332406.s009.tif]

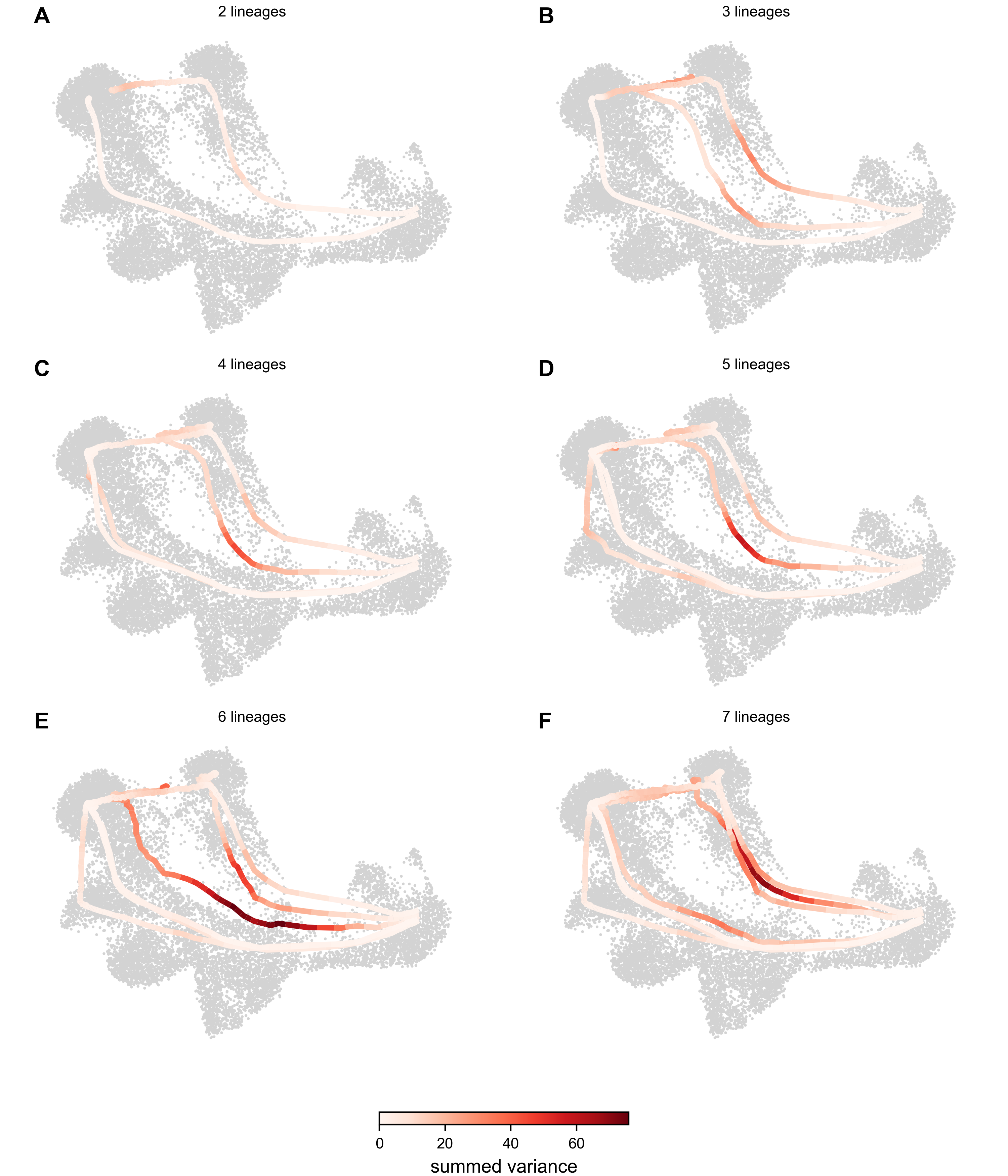

Supplement: S10 Fig — (A–F) Mean of inferred trajectory coordinates across 10 runs for different numbers of inferred lineages. The color scale represents the uncertainty in the form of the summed variance of 50 principal components across the runs. (TIF) [file pone.0332406.s010.tif]

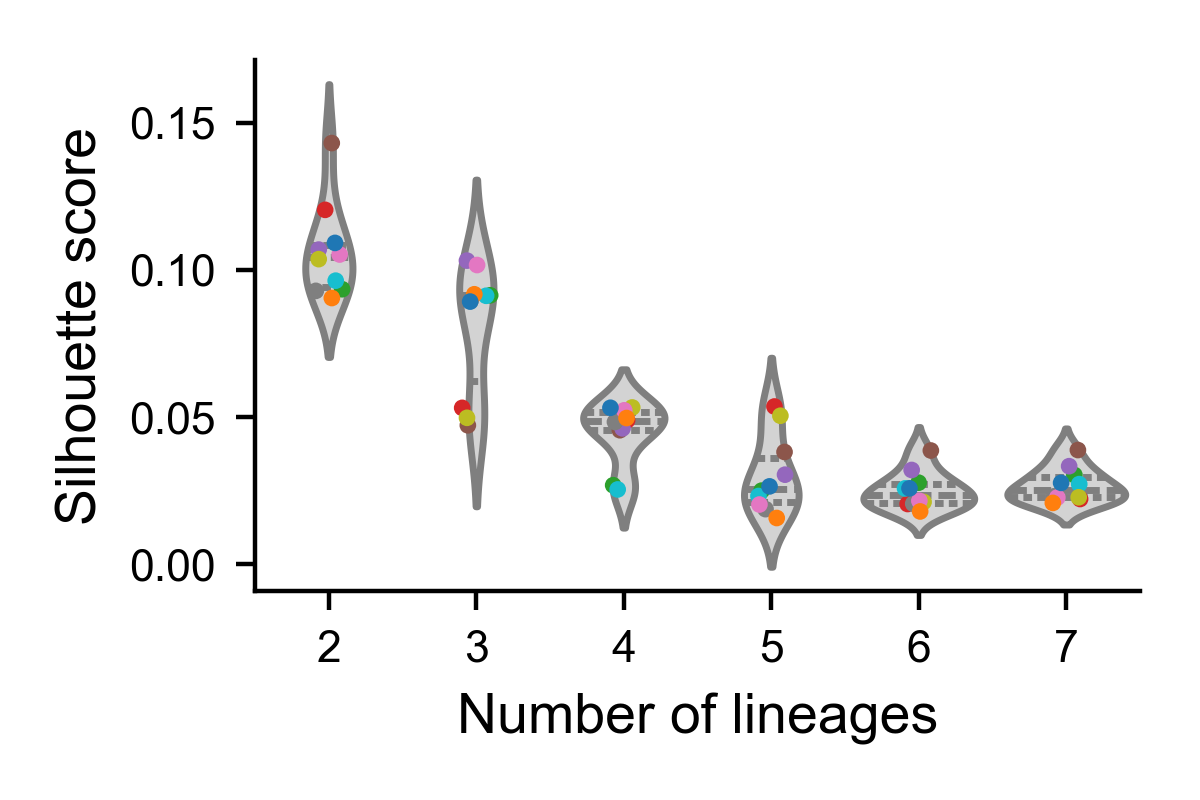

Supplement: S11 Fig — Colors represent individual simulation runs. (TIF) [file pone.0332406.s011.tif]

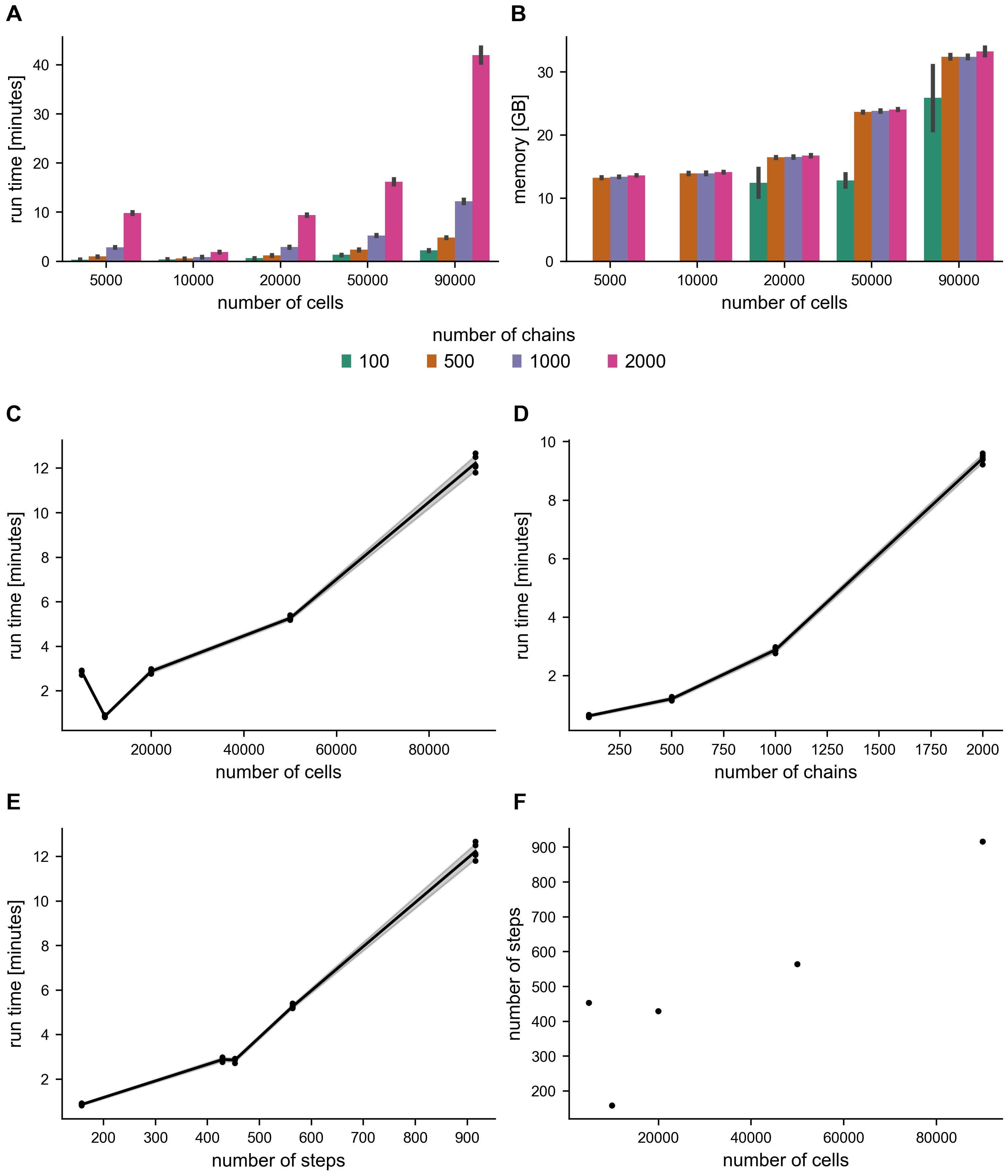

Supplement: S12 Fig — (A-B) Run time (A) and memory (B) requirements plotted versus the number of cells in the dataset, split by the number of simulated chains. Bars show the mean of five replicates. Error bars correspond to the standard deviation. (C) For a fixed number of chains (1,000), the run time rises approximately linearly with the number of cells. (D) For a fixed dataset size (20,000 cells), run time increases quadratically with the number of simulated chains. (E) Run time in relation to the number of simulated steps for a fixed number of chains (1,000). Lines and shaded regions in panels C-E show the mean and standard deviation of five replicates. (F) Relation between the number of cells in the dataset and the number of required simulation steps. (TIF) [file pone.0332406.s012.tif]

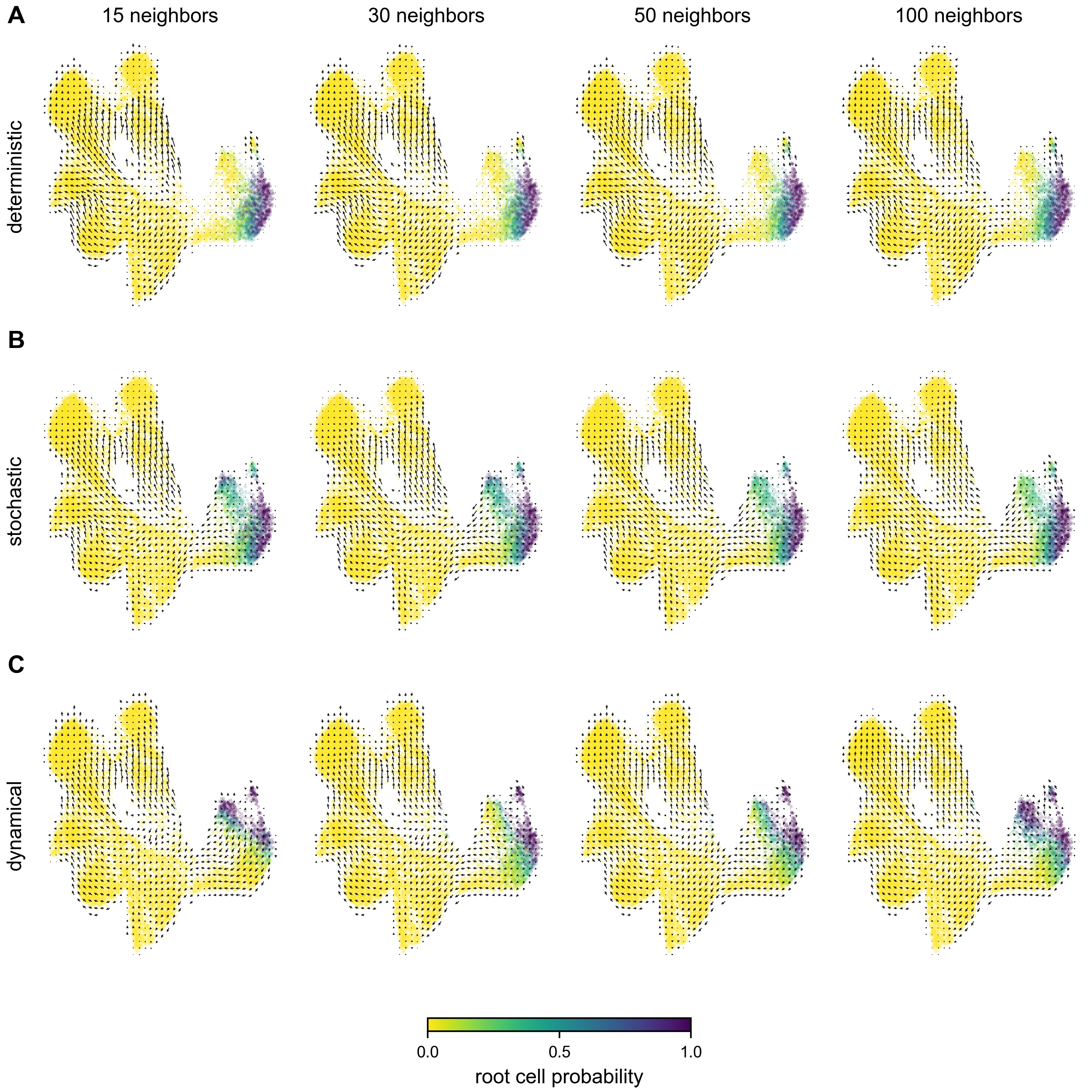

Supplement: S13 Fig — (A) Velocities computed with the deterministic model. (B) Velocities computed with the stochastic model. (C) Velocities computed with the dynamical model. Columns correspond (from left to right) to 15, 30, 50, and 100 nearest neighbors used for velocity computation. All subpanels show the inferred, embedded velocities smoothed on a grid. (TIF) [file pone.0332406.s013.tif]

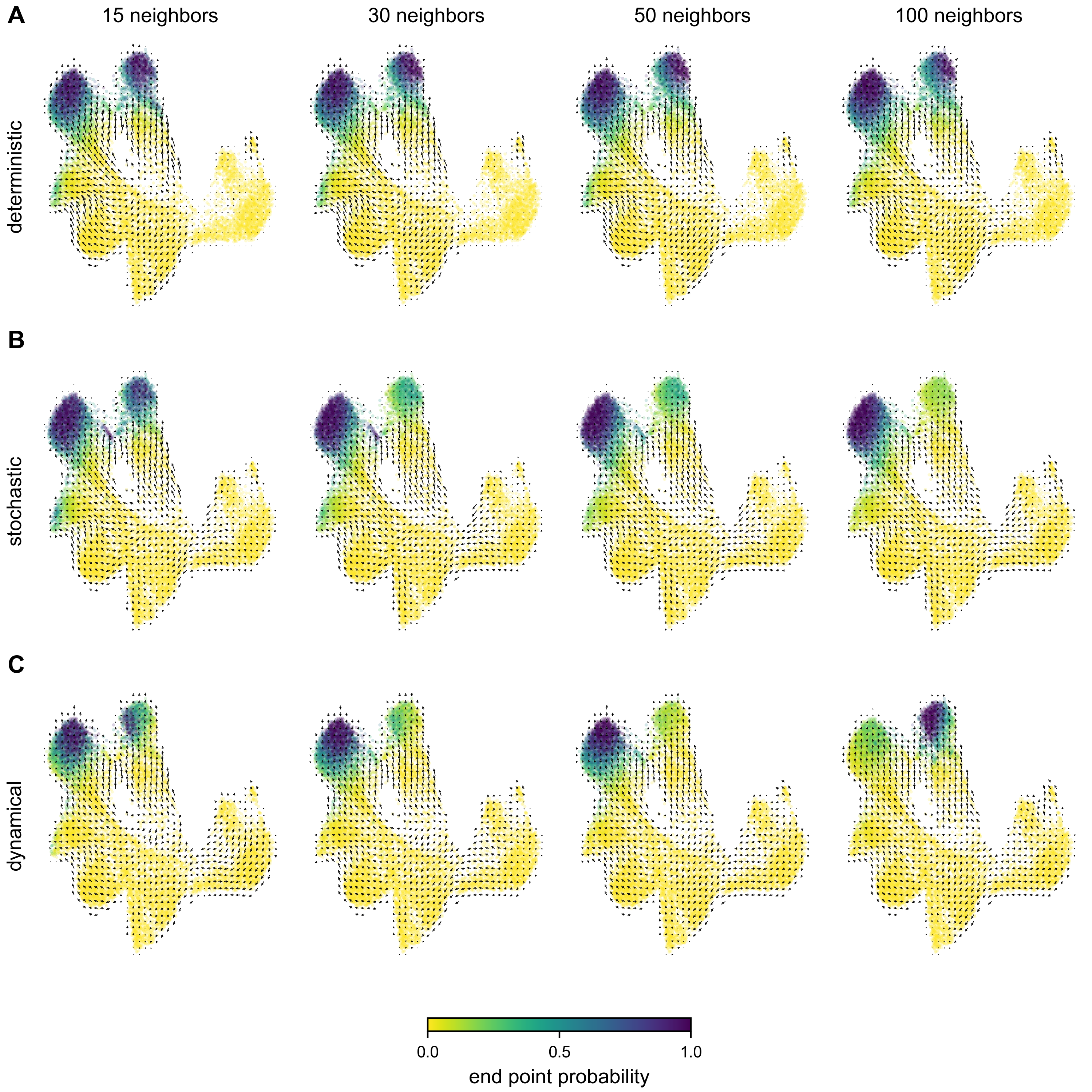

Supplement: S14 Fig — (A) Velocities computed with the deterministic model. (B) Velocities computed with the stochastic model. (C) Velocities computed with the dynamical model. Columns correspond (from left to right) to 15, 30, 50, and 100 nearest neighbors used for velocity computation. All subpanels show the inferred, embedded velocities smoothed on a grid. (TIF) [file pone.0332406.s014.tif]

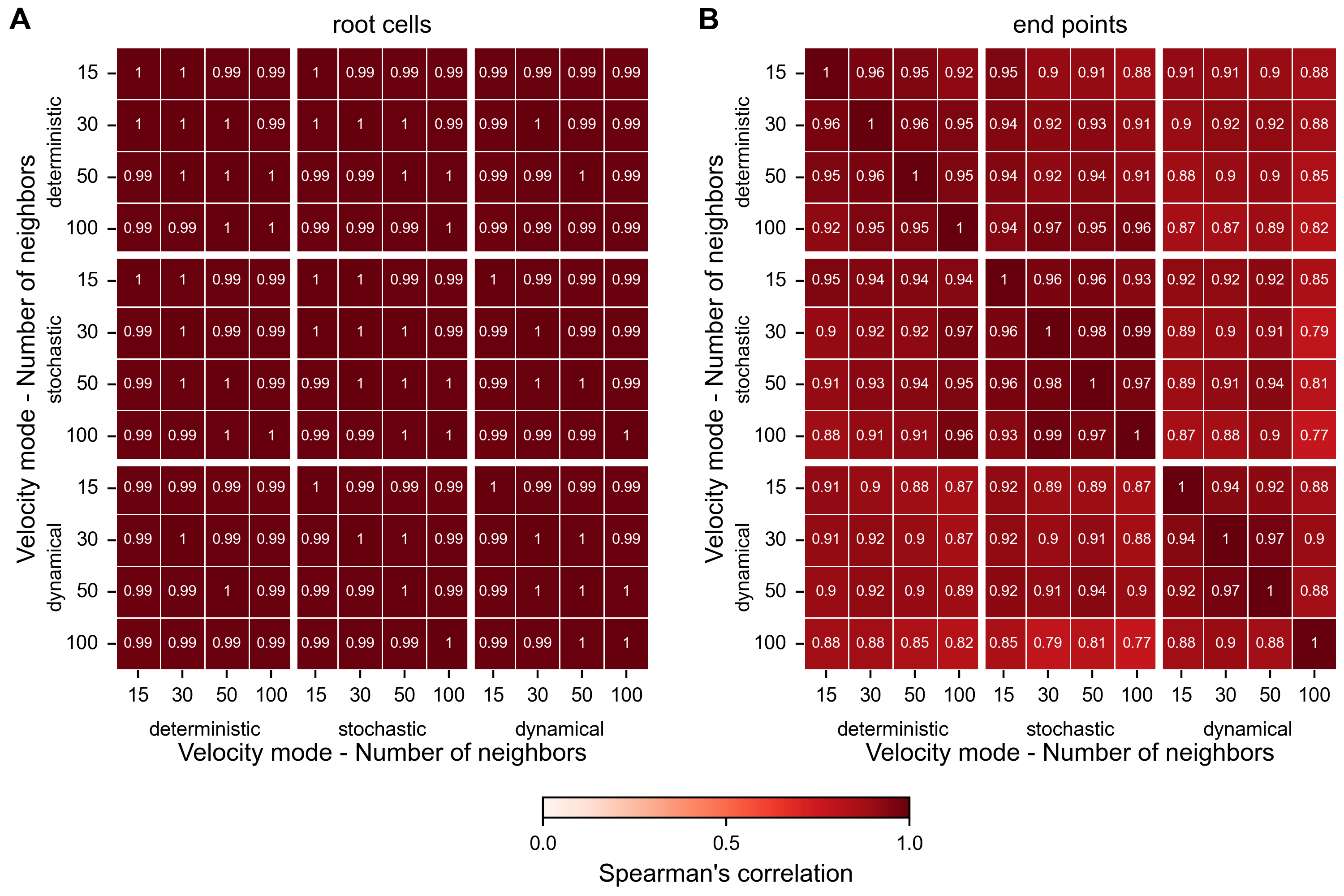

Supplement: S15 Fig — (A) Pairwise Spearman’s correlation between root cell probabilities. (B) Pairwise Spearman’s correlation between end point probabilities. Comparisons were made between the three modes of RNA velocity inference (deterministic, stochastic, and dynamical), and four different parameters for the number of neighbors (15, 30, 50, and 100 nearest neighbors). (TIF) [file pone.0332406.s015.tif]

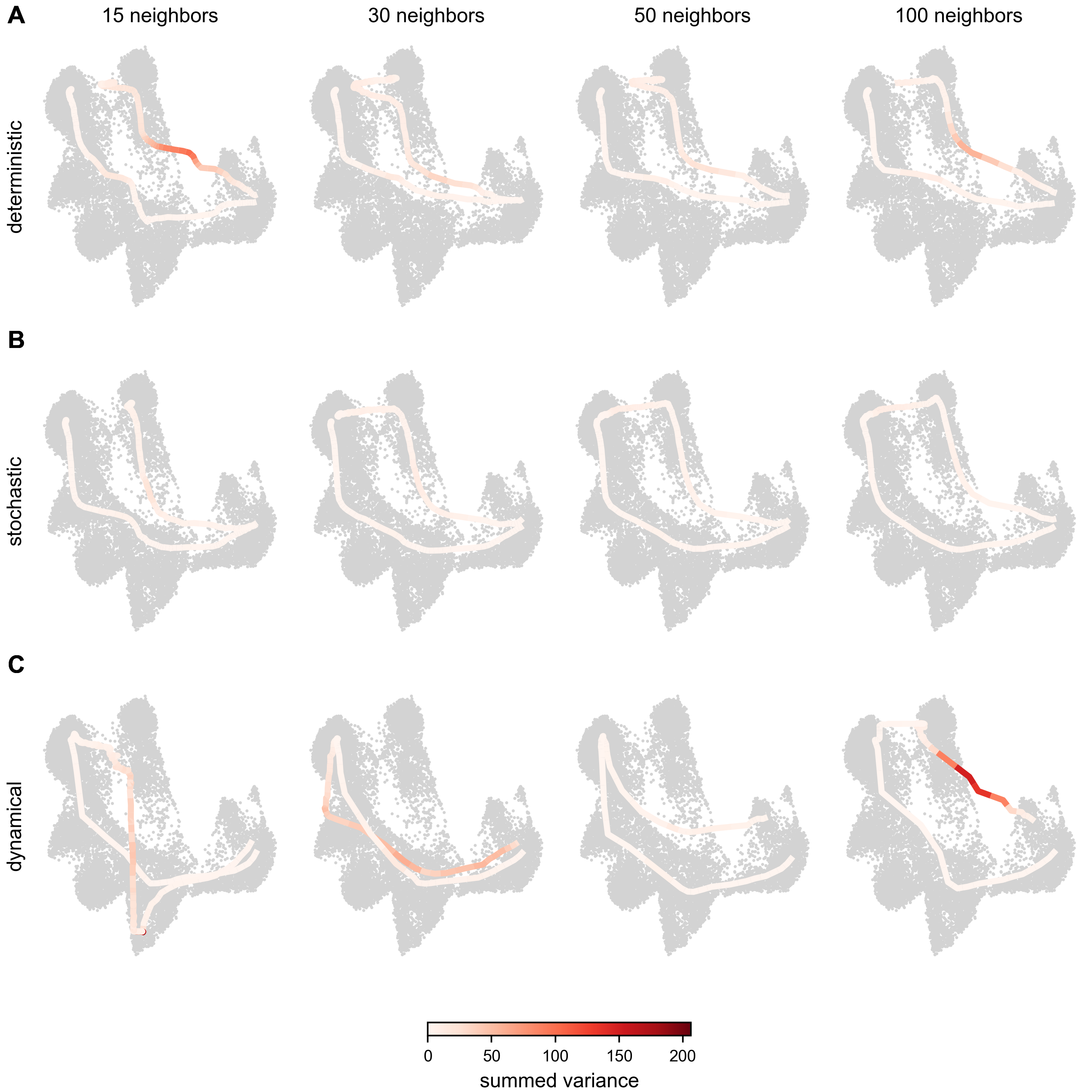

Supplement: S16 Fig — (A) Lineages based on velocities computed with the deterministic model. (B) Lineages based on velocities computed with the stochastic model. (C) Lineages based on velocities computed with the dynamical model. Columns correspond (from left to right) to 15, 30, 50, and 100 nearest neighbors used for velocity computation. Each panel shows the mean of inferred trajectory coordinates across 10 runs. The color scale represents the uncertainty in the form of the summed variance of 50 principal components across the runs. (TIF) [file pone.0332406.s016.tif]

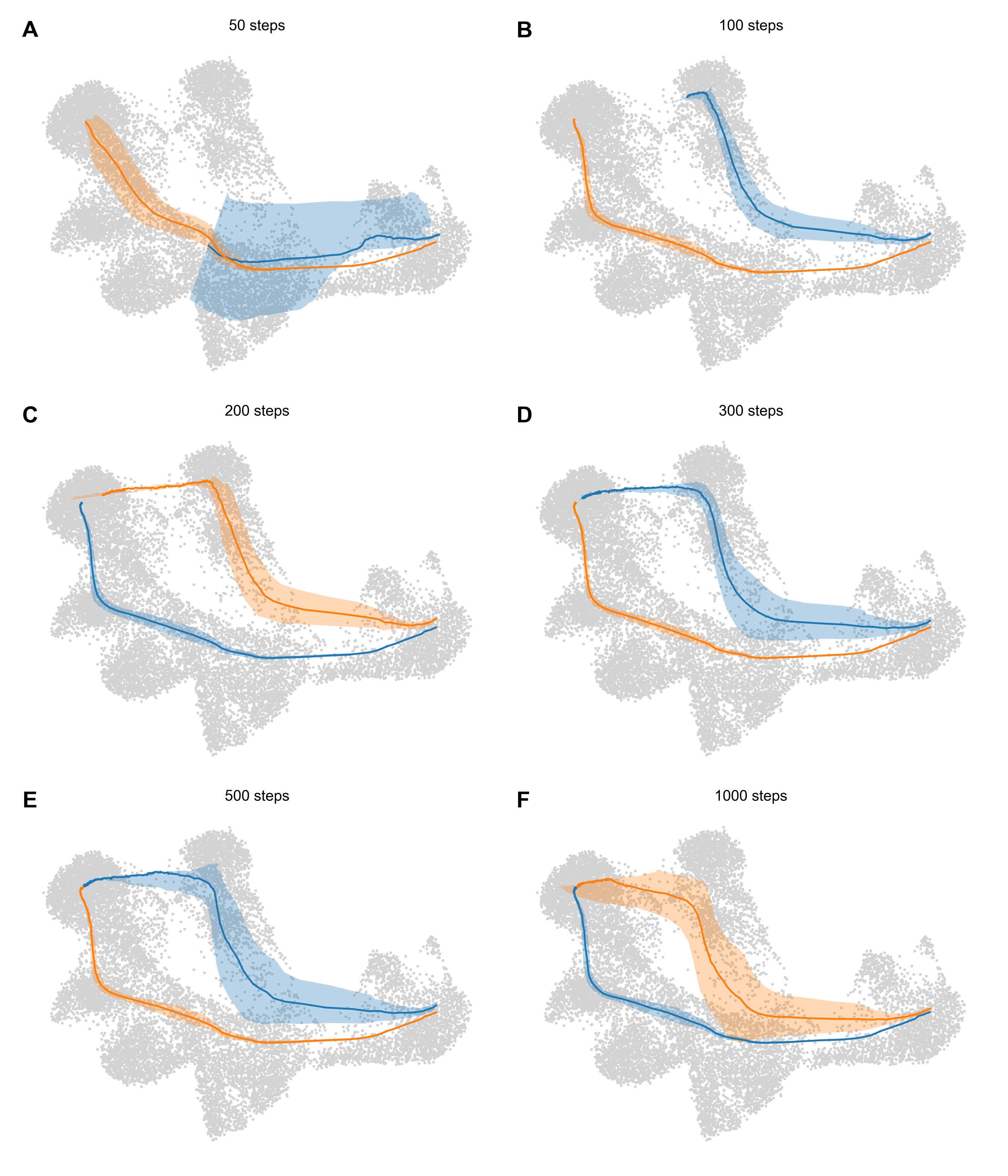

Supplement: S17 Fig — (A-F) Mean and standard deviation of inferred trajectory coordinates across 10 runs for different numbers of simulation steps. Each lineage is represented by a distinct color. (TIF) [file pone.0332406.s017.tif]

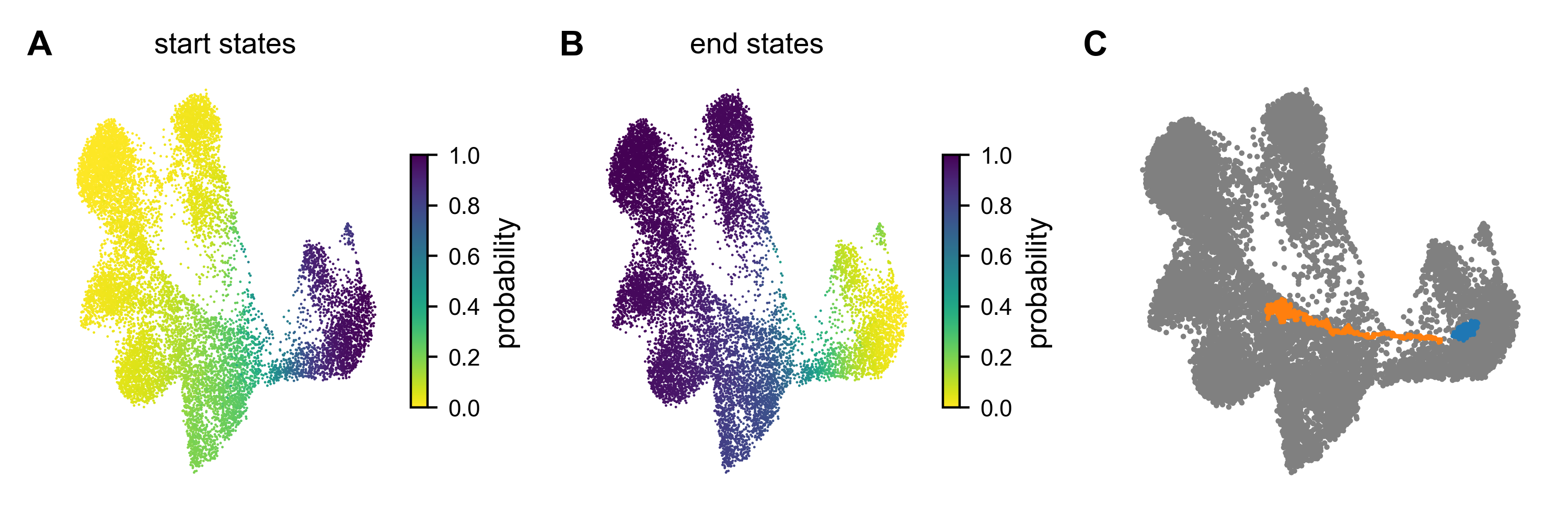

Supplement: S18 Fig — (A) Root cell probabilities based on connectivities. (B) End point probabilities based on connectivities. (C) Inferred trajectories based on the connectivity transition matrix. (TIF) [file pone.0332406.s018.tif]

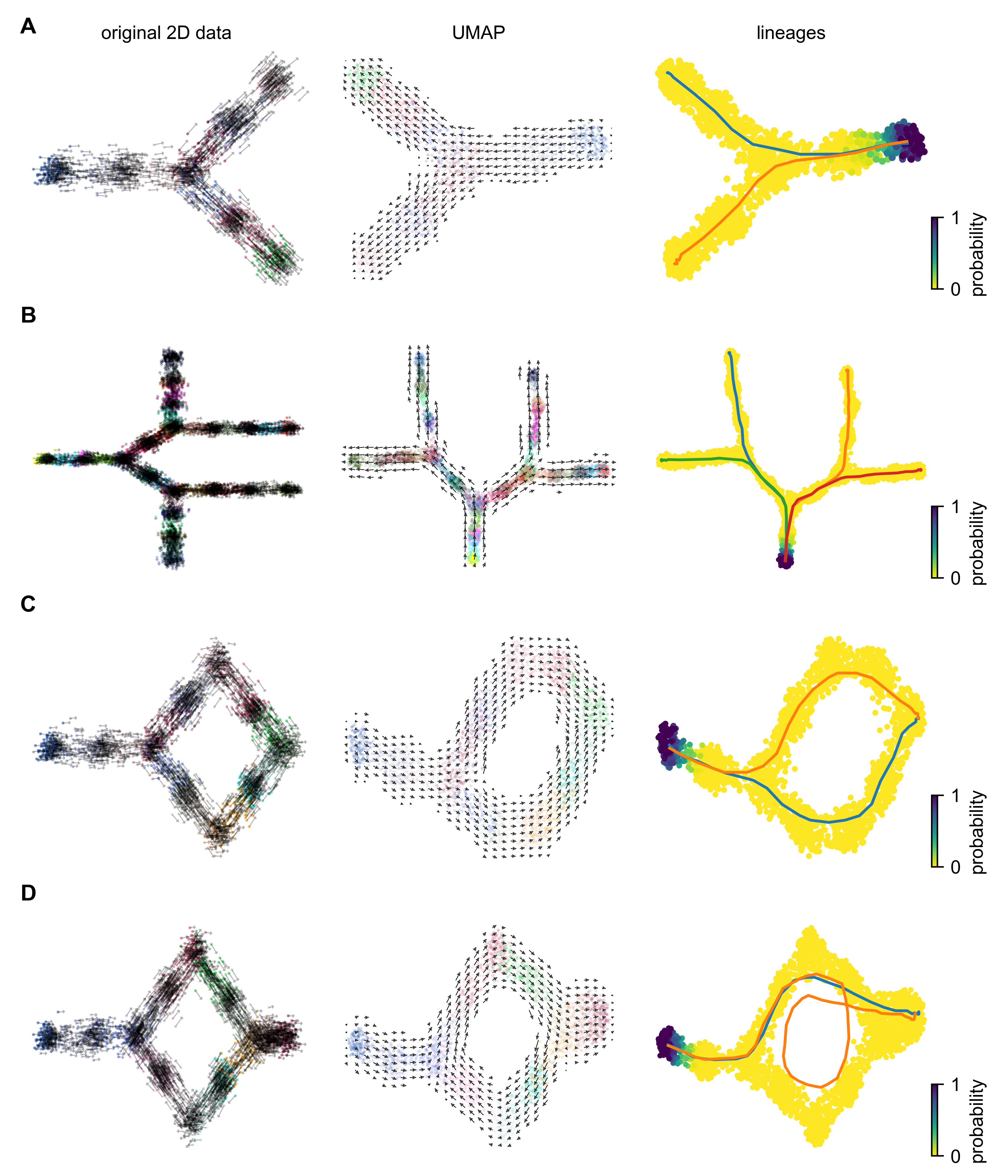

Supplement: S19 Fig — Data was generated in two dimensions with a bifurcating (A), double bifurcating (B), converging (C), and cyclical (D) topology. The left column shows the original, two-dimensional data and velocities, with points colored by their cluster of origin. The center column depicts velocities projected onto a UMAP embedding, with points colored by cluster. The right column shows lineages inferred with exploratory trajectory inference. Points are colored by their root cell probabilities to indicate trajectory directionality. (TIF) [file pone.0332406.s019.tif]

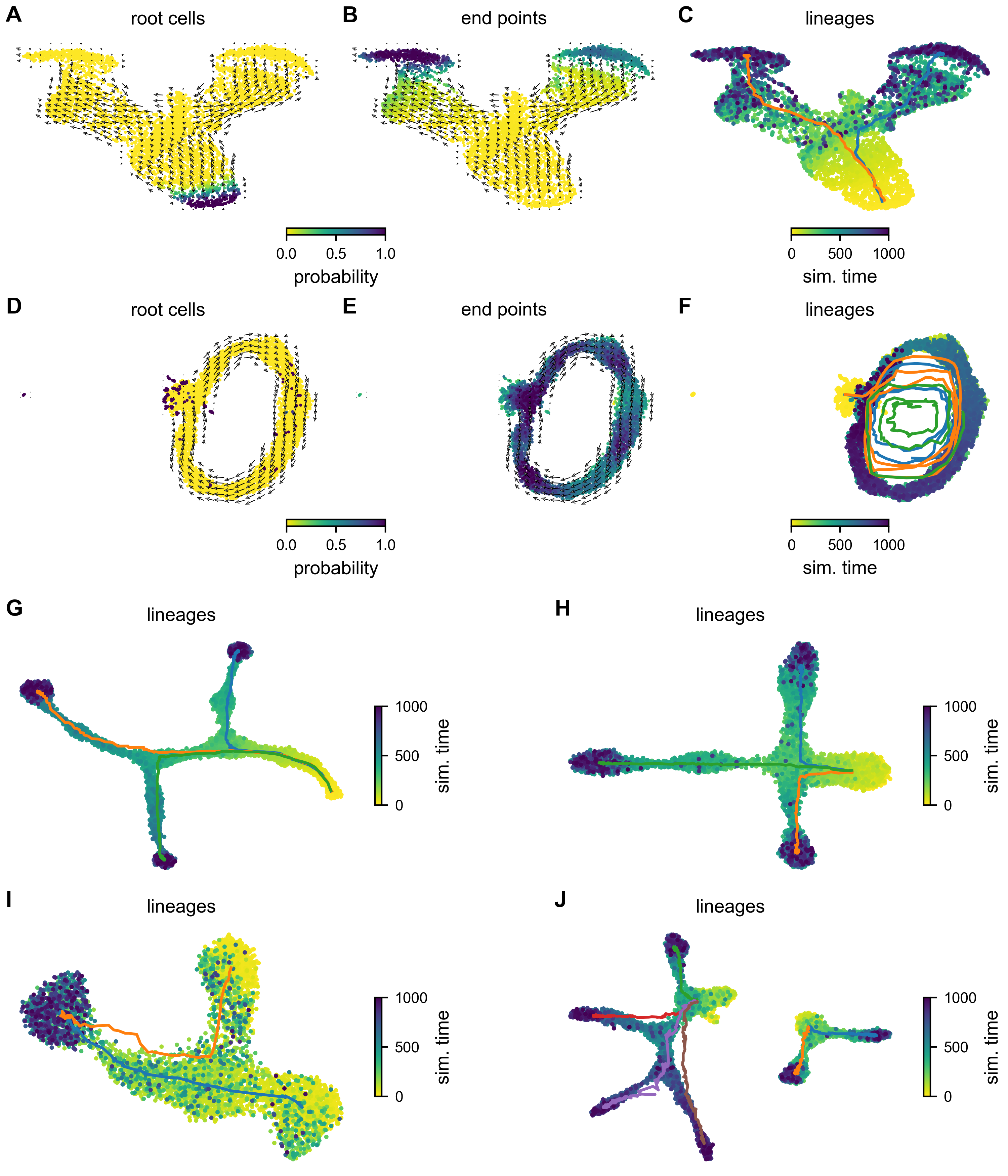

Supplement: S20 Fig — (A-C) Trajectory inference for dyngen’s bifurcating backbone. Velocities were projected onto a UMAP embedding, colored by root cell (A) and end point (B) probability. Inferred lineages are shown on the same embedding, colored by true simulation time (C). (D-F) Trajectory inference for dyngen’s cycle backbone. Velocities were projected onto a UMAP embedding, colored by root cell (D) and end point (E) probability. Inferred lineages are shown on the same embedding, colored by true simulation time (F). (G-J) Inferred lineages for the consecutive bifurcating (G), trifurcating (H), converging (I), and disconnected (J) dyngen backbone. Color represents ground-truth simulation time. (TIF) [file pone.0332406.s020.tif]

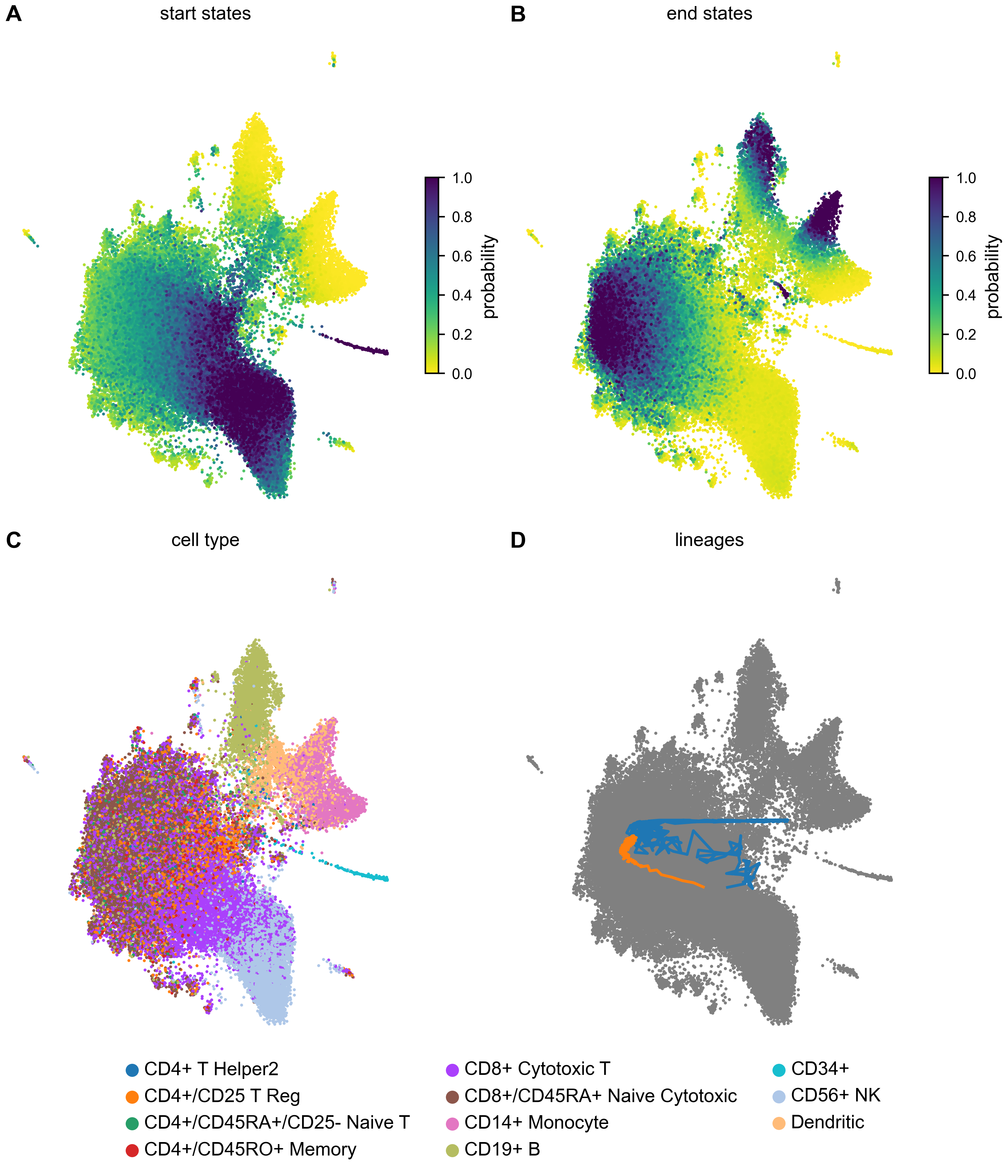

Supplement: S21 Fig — (A) Root cell probabilities based on RNA velocity. (B) End point probabilities based on RNA velocity. (C) Cell type annotations. (D) Lineages inferred with exploratory trajectory inference. (TIF) [file pone.0332406.s021.tif]

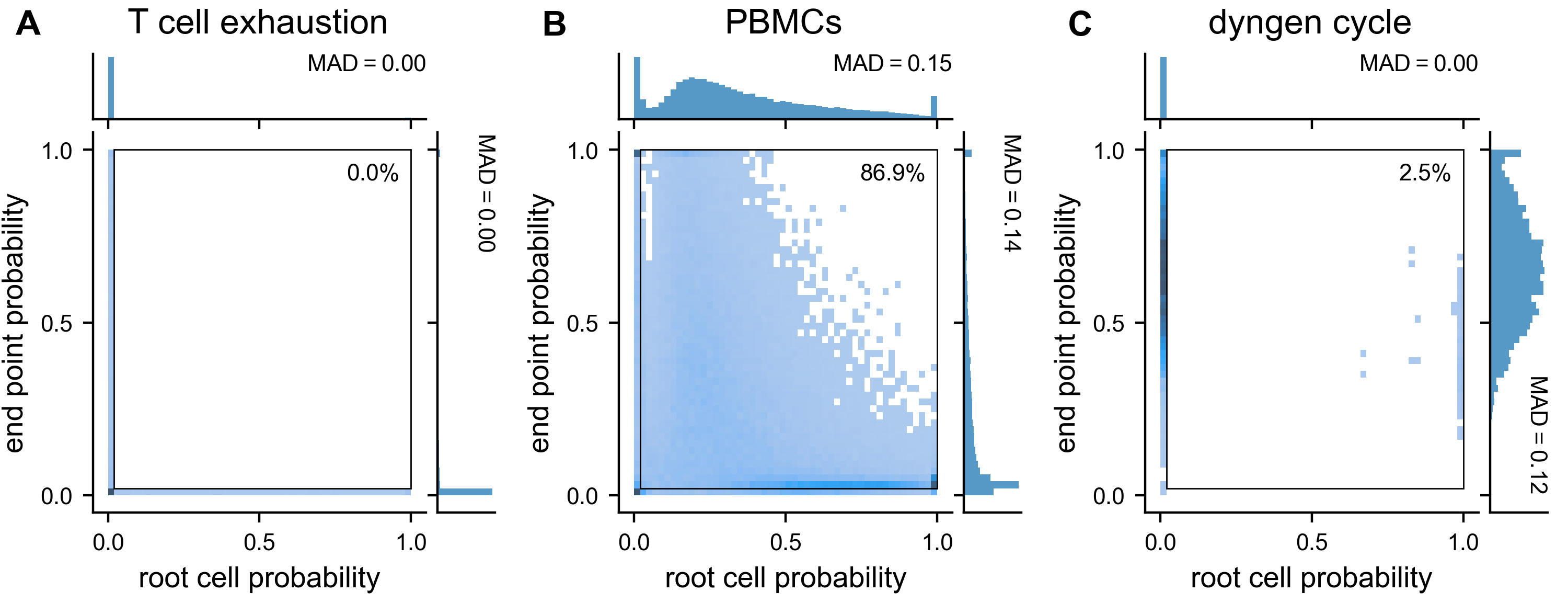

Supplement: S22 Fig — Joint and marginal distributions of root cell and end point probabilities for T cell exhaustion (A), PBMC (B), and dyngen cycle data (C). The black rectangle indicates cells with both non-negligible root cell and end point probability, with the percentage of cells falling into this area indicated in the top right corner. For the marginal distributions, median absolute deviation (MAD) is shown. (TIF) [file pone.0332406.s022.tif]
