# Supplementary material for: Exploratory trajectory inference reveals convergent lineages for CD8 T cells in chronic LCMV infection
Source: PLoS One. 2025 Sep 19;20(9):e0332406. doi: 10.1371/journal.pone.0332406 (PMC12448961; doi:10.1371/journal.pone.0332406)
Supplement: Attachment — Submitted filename: Rebuttal_Letter.pdf [file pone.0332406.s023.pdf]

Please find below the point-by-point response to the reviewer comments. Reviewer comments are copied in green font, author responses in black font, and text citations from the revised manuscript are displayed in black italic font. Reference numbers within the cited text refer to the manuscript references.

1. To show stability of root/terminal cell probabilities parameters over a range of nearest neighbors (Figure S12, S13) please plot the results in a more quantitative manner (e.g., pairwise correlations between root/terminal probabilities across hyperparameters, or the distributions of these probabilities over the hyperparameters). Otherwise readers need to interpret the grid of embeddings in a qualitative manner.

We acknowledge that our previous comparison of root cell and endpoint probabilities across hyperparameters used in RNA velocity inference relied on a qualitative interpretation of the grid of embeddings. According to the reviewer's suggestion, we have added a quantitative evaluation in the form of pairwise Spearman's correlations between probability assignments (S15 Fig). We find these are very similar across velocity estimation procedures and numbers of neighbors (median correlation of 0.99 and 0.91 for root cell and end point probabilities, respectively). We have adjusted the respective description in the Discussion section (lines 348-354):

> *For the three different models in scVelo ("deterministic", "stochastic", and "dynamical") and four different settings for the number of neighbors (15, 30, 50, and 100), the inferred velocities, root cells (S13 Fig), and end points (S14 Fig) were very similar, as confirmed by high pairwise Spearman's correlation (S15 Fig), and mostly differed in the position of the root cells within the early cluster and in the magnitude of end cell probabilities of the terminally exhausted and memory-like clusters.*

2. I believe the term 'diffuse' is used to define widely distributed root cell probabilities which may lead to harder to interpret trajectories. However, further (quantitative) discussion would be beneficial to make this property of use to users, to be able to assess their data. For example, is there some property of the transition matrix that demonstrate this diffuseness, or can this be plotted in the width/variance of inferred root cell probabilities?

We thank the reviewer for the question. To clarify this point, we have added a supplementary figure (S22 Fig) to the manuscript, alongside the following explanation in the Discussion section (lines 420-432):

> *To judge the diffuseness of terminal states, we suggest assessing the joint and marginal distributions of root cell and end point probabilities (S22 Fig). In a situation of low diffuseness of terminal states, the majority of cells has a root cell and end point probability of zero, few cells have a high probability for either state, the marginal distributions have a low median absolute deviation (MAD), and, most importantly, there are no cells with non-negligible probability for both terminal states. The T cell exhaustion dataset analyzed in this study exhibits the above properties indicative of a situation of low diffuseness of terminal states (S22 Fig A). For the PBMC dataset, we find a situation of high diffuseness, i.e., the majority of cells could, to a varying extent, be either a starting or an end state, and the marginal distributions have a higher MAD*

(S22 Fig B). The simulated dyngen cycle dataset lies between these two extremes, with well-defined root cells, but more widely spread end point probabilities (S22 Fig C).

3. Apologies for the potential confusion regarding the comment asking for uncertainty analysis of the trajectories generated over multiple runs. The intent was to assess the variance in the multiple realizations of trajectories in the system, which should be computed in whatever space the trajectories are created in (and clustered etc), which I believe is the 50d PCA space. Assessing variance in the UMAP coordinates adds the layer of a non-deterministic, nonlinear transformation and thus analysis of variance/noise also induced by that process not just through the variance in the trajectories themselves.

Thus this analysis should be performed in the original trajectory space (and be easily available for users to check).

It would also help to visualize variance in these paths in Figure S14, particularly if in scenarios where paths are not recovered correctly and we see more variance/uncertainty in the produced trajectories, i.e., if there is anything a user could use to tell how much to trust or choose between those outputs.

According to the reviewer's suggestion, we have added an assessment of the variance in inferred trajectories over multiple runs in the 50-dimensional PCA space. For an easily interpretable visualization, we represent this variance for each step of the trajectory by the color of the line showing the mean inferred trajectory coordinate across multiple runs in the UMAP embedding (S10 Fig). We performed the same analysis for the experiments with varying RNA velocity estimation parameters (S16 Fig, formerly S14 Fig), where previously the results of a single execution of exploratory trajectory inference were shown.

The Materials and Methods subsection "Uncertainty estimation of inferred trajectories" was updated to reflect this change (lines 588-592):

> *The mean and standard deviation of trajectory coordinates in UMAP space, as well as the variance of coordinates in PCA space across the repeated mapped runs, were used to visualize the uncertainty of inferred lineages on the UMAP embedding. For the latter, we visualized the sum of the variance of all 50 principal components.*

The results for different numbers of lineages are reported in the Results section, lines 218-220:

> *We found increasing uncertainty and overlap between lineages with an increasing number of lineages. The same trend was observed in PCA space in the form of the variance for each step across 10 runs, summed over all 50 principal components (S10 Fig).*

For the results with varying velocity estimation parameters, the Discussion section was adapted as follows (lines 356-364):

Notably, the only qualitative difference between the trajectories inferred from stochastic velocities across four different neighborhood sizes was the failure to detect the transition from memory-like to terminally exhausted cells with 15 neighbors. The dynamical model resulted in larger deviations for 15 and 30 neighbors, but produced similar qualitative trajectories for 100 neighbors. However, for the two strongly deviating results based on the dynamical model, the inferred trajectories also showed a higher variance across repeated runs than those for the stochastic model. Our findings reported in this study, obtained with the stochastic model and 30 nearest neighbors, were supported by the majority of trajectory inference analyses with the other considered parameters.

4. For the simulations of the different topologies, please provide details on how many cells/observations were sampled from this model. This Gaussian model also does not represent sparse single-cell count data so the authors should comment on these assumptions (how this may influence results) and demonstrate that the generated points provided to scVelo resemble at least the magnitude of values usually provided in single-cell data.

Additionally, why was a count-based simulator, like dyngen https://github.com/dynverse/dyngen for example, not used here to generate different topologies that also provide ‘realistic’ RNA counts (the usual input to scVelo)?

We have expanded our description of the data generation in the Materials and Methods section to indicate the number of generated observations (lines 636-642):

To generate positive control data with simple, well-known ground truth trajectories of varying topology, we constructed trajectories in two dimensions (see left column in S19 Fig). To this end, we defined cluster means forming the desired topology and sampled 100 points from a bivariate Gaussian distribution around the means. Additionally, we sampled interpolated points lying between the main clusters (100 points for each pair of consecutive clusters). This resulted in 1,300, 3,700, 2,000, and 2,400 data points for the bifurcating, double bifurcating, converging, and cyclical topologies, respectively.

We acknowledge that these simulations represent a strong simplification and do not represent the statistical properties of single-cell RNA sequencing data. According to the reviewer’s comment, we have now included dyngen to generate more realistic simulated data, as discussed in lines 387-405.

Specifically, we simulated data from processes constructed in 2D (see Simulation of positive control data for details on data generation). [...] While showcasing the capability of our method to infer lineages in situations with velocities strongly aligned with the defined dynamic process, it does not represent the statistical properties of sparse scRNAseq count data. Therefore, to generate more realistic simulations with known trajectories, we additionally used dyngen [29], with various backbones, resulting in diverse process topologies. Here, exploratory trajectory inference showed overall good performance in recovering the true trajectories as well (S20 Fig). Automatic selection yielded the correct number of lineages for all backbones except

for the cycle. Here, we obtained three lineages instead of one, all following the cyclic dynamic process. However, this simulated dataset was particularly challenging since the inferred end points are distributed all along the cyclic path, and the ground truth simulation also showed multiple cycles (S20 Fig D-F), in contrast to, e.g., the bifurcating backbone, where end points were more restricted and where there were no cyclical patterns (S20 Fig A-C).

The data generation with dyngen is described in detail in the Materials and Methods section, lines 651-666:

Additional positive control datasets were generated with dyngen [29] in R using the bifurcating, cycle, consecutive bifurcating, trifurcating, converging, and disconnected backbones. For each dataset, 500 genes were simulated for 5,000 cells. The number of transcription factors was set to the number of modules in the respective backbone. Half of the remaining genes were simulated as targets, the rest as housekeeping genes. The simulation parameters were set as follows: total_time = 1000, census_interval = 2, ssa_algorithm = ssa_etl(tau = 300 / 3600). We performed wild-type simulations with 500 simulations for a snapshot experiment, computing RNA velocity as well. The data was further processed using Scanpy and scVelo, using the simulated counts, spliced counts, unspliced counts, and RNA velocity. Genes with less than 20 shared counts (spliced and unspliced) were removed, and the three count matrices were normalized to the respective median of total counts. The expression data was log-transformed and scaled. PCA, a neighborhood graph, and a UMAP projection were computed with default parameters. Normalized spliced and unspliced counts were smoothed using scvelo.pp.moments, and a velocity graph was built using neighbor connectivities. Terminal states and a transition matrix were computed.
